# Human Leukocyte Antigen Markers for Distinguishing Pustular Psoriasis and Adult-Onset Immunodeficiency with Pustular Reaction

**DOI:** 10.3390/genes15030278

**Published:** 2024-02-23

**Authors:** Apiwat Sangphukieo, Patcharawadee Thongkumkoon, Pitiporn Noisagul, Luca Lo Piccolo, Timothy E. O’Brien, Suteeraporn Chaowattanapanit, Charoen Choonhakarn, Warayuwadee Amornpinyo, Romanee Chaiwarith, Salin Kiratikanon, Rujira Rujiwetpongstorn, Napatra Tovanabutra, Siri Chiewchanvit, Piranit Kantaputra, Worrachet Intachai, Sivamoke Dissook, Mati Chuamanochan

**Affiliations:** 1 Center of Multidisciplinary Technology for Advanced Medicine (CMUTEAM), Faculty of Medicine, Chiang Mai University, Chiang Mai 50200, Thailand; apiwat.sang@cmu.ac.th (A.S.); patcharawadee.t@cmu.ac.th (P.T.); pitiporn.noi@cmu.ac.th (P.N.); lopiccolo.l@cmu.ac.th (L.L.P.); 2 Applied and Environmental Statistics, Department of Mathematics and Statistics, Loyola University Chicago, Chicago, IL 60153, USA; tobrie1@luc.edu; 3 Division of Dermatology, Department of Medicine, Faculty of Medicine, Khon Kaen University, Khon Kaen 40002, Thailand; csuteeraporn@yahoo.com (S.C.); c_choonhakarn@yahoo.com (C.C.); 4 Division of Dermatology, Department of Internal Medicine, Khon Kaen Hospital, Ministry of Public Health, Khon Kaen 40002, Thailand; warayuwadee.a@gmail.com; 5 Division of Infectious Diseases and Tropical Medicine, Department of Internal Medicine, Faculty of Medicine, Chiang Mai University, Chiang Mai 50200, Thailand; rchaiwar@gmail.com; 6 Division of Dermatology, Department of Internal Medicine, Faculty of Medicine, Chiang Mai University, Chiang Mai 50200, Thailand; s.kiratikanon@gmail.com (S.K.); rujira.r.330@gmail.com (R.R.); ntovanabutra@gmail.com (N.T.); drsiri2010@gmail.com (S.C.); 7 Center of Excellence in Medical Genetics Research, Chiang Mai University, Chiang Mai 50200, Thailand; dentaland17@gmail.com (P.K.); worrachet.intachai@gmail.com (W.I.); 8 Division of Pediatric Dentistry, Department of Orthodontics and Pediatric Dentistry, Faculty of Dentistry, Chiang Mai University, Chiang Mai 50200, Thailand; 9 Department of Biochemistry, Faculty of Medicine, Chiang Mai University, Chiang Mai 50200, Thailand

**Keywords:** adult-onset immunodeficiency, anti-interferon γ autoantibodies, human leukocyte antigen, pustular psoriasis, pustular reaction, pustular skin diseases

## Abstract

Pustular skin diseases, with pustular psoriasis (PP) being the prototype, are immune-mediated diseases characterized by the presence of multiple pustules, resulting from neutrophil accumulation in the layer of epidermis. Sterile skin pustular eruption, like PP, is also observed in 20–30% of patients with adult-onset immunodeficiency syndrome (AOID) and anti-interferon γ autoantibodies (IFN-γ), leading to challenges in classification and diagnosis. While the mechanism underlying this similar phenotype remains unknown, genetic factors in relation to the immune system are suspected of playing an important role. Here, the association between human leukocyte antigen (HLA) genes, which play essential roles in antigen presentation, contributing to immune response, and the presence of skin pustules in AOID and PP was revealed. HLA genotyping of 41 patients from multiple centers in Thailand who presented with multiple sterile skin pustules (17 AOID patients and 24 PP patients) was conducted using a next-generation-sequencing-based approach. In comparison to healthy controls, HLA-B*13:01 (OR = 3.825, 95%CI: 2.08–7.035), C*03:04 (OR = 3.665, 95%CI: 2.102–6.39), and DQB1*05:02 (OR = 2.134, 95%CI: 1.326–3.434) were significantly associated with the group of aforementioned conditions having sterile cutaneous pustules, suggesting a common genetic-related mechanism. We found that DPB1*05:01 (OR = 3.851, *p* = 0.008) and DRB1*15:02 (OR = 3.195, *p* = 0.033) have a significant association with pustular reaction in AOID patients, with PP patients used as a control. A variant in the *DRB1* gene, rs17885482 (OR = 9.073, *p* = 0.005), was observed to be a risk factor for PP when using AOID patients who had pustular reactions as a control group. DPB1*05:01 and DRB1*15:02 alleles, as well as the rs17885482 variant in the *DRB1* gene, were proposed as novel biomarkers to differentiate PP and AOID patients who first present with multiple sterile skin pustules without known documented underlying conditions.

## 1. Introduction

**Figure 1:**
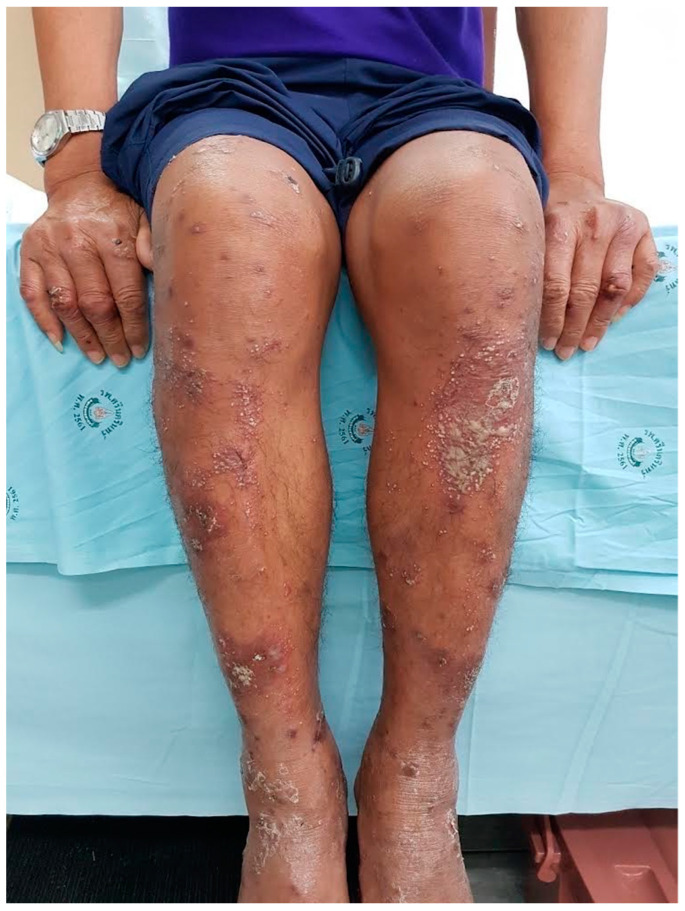
Multiple sterile non-follicular pustules arising in erythematous patches; some coalesce to form lakes of pus on dorsal aspect of both hands and both lower extremities in patient with adult-onset immunodeficiency syndrome (AOID) due to anti-interferon γ autoantibodies. The morphology of this pustular reaction is similar to other pustular skin diseases, e.g., generalized pustular psoriasis [17].

Pustular psoriasis (PP) and related pustular skin disorders are immune-mediated diseases consisting of several clinical entities including localized pustular psoriasis, mainly palmoplantar pustular psoriasis (PPP) and acrodermatitis continua of Hallopeau (ACH), generalized pustular psoriasis (GPP), a life-threatening form of psoriasis, and acute exanthematous generalized pustular eruption (AGEP), a severe subtype of drug hypersensitivity [1]. These entities are recognized by the presence of multiple pustules as a result of extensive neutrophilic accumulation [1,2,3]. The sterile pustular eruptions noted in 20–30% of patients with adult-onset immunodeficiency (AOID) are particularly noteworthy. AOID is an acquired form of immunodeficiency, occurring in adults who previously had normal immune function. This condition arises due to the development of autoantibodies against interferon-γ (IFN-γ), a crucial cytokine in immune response. The resultant immunodeficiency in AOID patients manifests similarly to AIDS, with patients being susceptible to a range of opportunistic infections (OIs) typically seen in those with severely compromised immune systems [4]. This phenomenon has been extensively studied, with reactive neutrophilic dermatoses being identified as a common feature in AOID due to the anti-IFN-γ autoantibody [2]. Furthermore, a variety of reactive and infective dermatoses, including Sweet’s syndrome, have been associated with AOID [5]. Since this pustular reaction in AOID is phenotypically similar to other pustular skin disorders (Figure 1), the terms “PP”, “AGEP”, “exanthematous pustulosis”, “generalized pustular eruption”, and “subcorneal pustulosis” have been used variably and interchangeably in this condition [3,4,6,7,8,9].

While the biological mechanism of this similar phenotype remains unknown, both AOID and PP are thought to be multifactorial diseases involving pathogenic mutations in multiple genes in combination with environmental factors that could constitute the initial trigger of inappropriate immunity activation. To the best of our knowledge, genetic abnormalities in *IL36RN*, *CARD14*, and *AP1S3* genes have been proposed to play an important role in pathophysiological features in PP, but the high genetic heterogeneity of the diseases leads to ambiguous conclusions [1]. It has been estimated that the abnormalities in *IL36RN*, *CARD14*, and *AP1S3* account for fewer than 30% of the PP cases; therefore, additional genetic factors of the diseases remain to be elucidated [10]. Recently, a missense mutation in the predisposing gene, *SERPINA1*, has been identified in two unrelated patients, one with AOID who has pustular skin reaction and the other with PP, providing the first evidence of the crucial link between these two diseases [11].

A few genetic variations of the human leukocyte antigen (HLA), a set of genes encoding for cell-surface molecules responsible for the regulation of the immune system, either class I or II, have been identified in association with PP in the Japanese population [12,13], while *HLA* allele variants, mainly of class II, such as DRB1*16:02 and DQB1*05:02, have been associated with AOID in Taiwanese [14], Chinese [15], and more recently, Thai [16] populations. Despite these observations suggesting that HLA genes could play a critical role in the pathogenic mechanism of both AOID and PP, the role of HLA in the pustule formation remains unknown, so far. In this study, we performed the HLA genotyping of a Thai nationality cohort presenting multiple sterile skin pustules and compared it with the available dataset of a healthy unrelated Thai population to assess the genetic burden of HLA variants in the manifestation of these pustular skin diseases.

## 2. Materials and Methods

### 2.1. Study Subjects

A multicenter retrospective study was conducted from January 2005 to June 2020 using patients with pustular reaction in AOID and pustular psoriasis at Maharaj Nakorn Chiang Mai Hospital, Khon Kaen University’s Srinagarind Hospital, and Khonkaen Hospital, Thailand.

Diagnosis of AOID is confirmed by meeting the following conditions: a medical history showing OIs indicative of a Cell-Mediated Immunity (CMI) defect; ruling out other causes of immunosuppression such as Human Immunodeficiency Virus (HIV) infection, cancer, or the use of immunosuppressive medication; and the presence of antibodies against IFN-γ, verified through an enzyme-linked immunosorbent assay (ELISA).

The pustular reaction in AOID patients had been confirmed by clinical manifestation of multiple tiny non-follicular pustules with the exclusion of infection by either of the following microbiologic methods: (i) staining; (ii) culture; or (iii) molecular techniques. All participants denied a family and personal history of psoriasis. No culprit drugs were identified for acute generalized exanthematous pustulosis at the onset of the pustular eruption.

Pustular psoriasis was clinically diagnosed by a board-certified dermatologist and categorized into the generalized form (i.e., generalized PP) and the localized form, including palmoplantar pustulosis (PPP) and acrodermatitis continua of Hallopeau (ACH).

### 2.2. Whole-Exome Sequencing and Variant Calling for HLA Genes

Whole-exome sequencing was performed on exon targets isolated from genomic DNA from peripheral blood leucocytes. The DNA sample was prepared as an Illumina sequencing library enriched by SureSelect Human All Exon V6 Kit + UTR post (Illumina, San Diego, CA, USA) and was sequenced onto Illumina sequencing platform. The Burrows–Wheeler Aligner (BWA) version 07.17(r1188) was applied to align raw read data to human reference genome version 38 (hg38 without ALT contigs).

HLA genotyping was carried out as shown in Erick C. Castelli’s protocol version 2.0 [18] Briefly, hla-mapper and GATK4 HaplotypeCaller were applied to obtain unbiased read mapping and to name all possible HLA variants, respectively. Then, the variant output file was recorded and filter out variant artifacts using vcf tools version 0.1.16. The multi-allelic file was normalized to biallelic VCF file using bcftools (unphased genotype file) and used as input file in the association study. The identified variants were annotated using Ensemble VEP (https://www.ensembl.org/vep) accessed on 27 January 2022, ClinVar (https://www.ncbi.nlm.nih.gov/clinvar) accessed on 27 January 2022, dbSNP (https://www.ncbi.nlm.nih.gov/snp; accessed on 27 January 2022), gnomAD v3.1.2 (https://gnomad.broadinstitute.org; accessed on 11 March 2022), and 1000G (https://www.internationalgenome.org; accessed on 5 Febuary 2022).

### 2.3. HLA Typing

Four-digit HLA typing from short-read sequences was carried out using xHLA algorithm [19]. BWA was applied to align raw read data to human reference genome hg38 without ALT contigs to generate BAM files, which can be used as inputs for xHLA. xHLA was run for each sample under docker environment with command docker run humanlongevity/hla --sample_id ID --input_bam_path INPUT.bam --output_path OUTPUT.

### 2.4. HLA Allele and Haplotype Association Analysis

HLA allele and haplotype frequencies were compared between the patient group and Thai population as a control group. HLA allele and haplotype frequencies of Thai population were collected from two different sources reported in 2018 and 2020. The first paper [20] reported HLA class I and II diversity in 334 unrelated donors from a dengue vaccine efficacy trial conducted in the Muang District, Ratchaburi Province, Thailand, by using long-range PCR amplification and next-generation sequencing. The second paper reported the diversity of Thai HLA alleles on both HLA class I and II genes from 470 unrelated healthy individuals by using polymerase chain reaction sequence-specific oligonucleotide [21]. The latter dataset randomly chose the subjects according to their self-reported origins from five regions of Thailand, i.e., Bangkok (*n* = 70), Central (*n* = 100), Northeastern (*n* = 100), Northern (*n* = 100), and Southern (*n* = 100) parts. The relationship between an HLA haplotype and a disease status was quantified by odds ratio. The odds ratio was calculated using the number of patients who did or did not carry particular HLA type and the number of controls who did or did not carry such HLA type [22].

### 2.5. HLA Variant Enrichment and Association Analysis

Fisher’s exact test was performed to identify the set of HLA variants that are enriched in the patient group by using the East Asian (EAS) variants from 1000G project database [23] and gnomAD database [24]. HLA variants with frequency lower than 1% in the patient dataset were excluded from the study. Then, Bonferroni correction approach was applied to *p*-value to correct for multiple testing. Only the variants with adjusted *p*-value lower than 0.05 in both comparisons (1000G and gnomAD) were considered as significantly enriched in this dataset.

Association between candidate variants and pustular skin reaction and AOID patients were also observed. The candidate variants were analyzed by using a logistic regression model adjusted for gender and age to test the association between AOID and no AOID (pustular skin reaction) status as controls using PLINK. Empirical *p*-value was obtained from *max(T)* method with 10,000 permutations. The candidate variants’ pairwise linkage disequilibrium statistics (D′ and R2) were calculated using LDmatrix Tool (https://ldlink.nci.nih.gov/?tab=ldmatrix; accessed on 10 February 2023) with EAS population as a reference and PLINK software (Version 1.9).

### 2.6. Structural Modeling

The sequences of HLA-B, C, DQB1, DPB1, and DRB1 were extracted from human reference genome GRCh38 and submitted to SWISS-MODEL [25], an automated protein-structure-homology-modeling server with default parameters. The protein structure of 6AT5, 5VGE, 7KEI 4P57, and 4X5W from Protein Data Bank (PDB) was selected as a template based on sequence identity for HLA-B, C, DQB1, DPB1, and DRB1 modeling, respectively. The α chain sequences of HLA-DQA1 (UniprotKB P01909) and HLA-DRA1 (UniprotKB P01903) were used as a heterodimer to construct the complete structure of HLA-DQ and HLA-DR complexes, respectively. The protein structures were visualized using Pymol (http://www.pymol.org/pymol; accessed on 1 May 2023).

## 3. Results

### 3.1. Demographic Characteristics of Pustular Disease Patients

**Figure 2:**
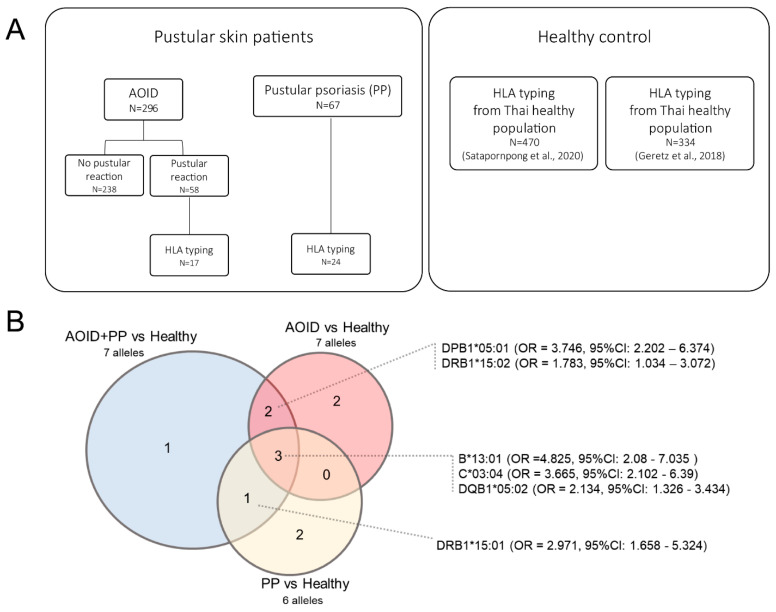
Study flow diagram of patients with pustular skin diseases and healthy control (**A**). Venn diagram demonstrates overlapped significant alleles of different association analysis conditions (**B**). The overlapped significant alleles are labeled with odds ratio and 95% confidence interval of AOID and PP versus healthy individuals.

In the cohort of 296 patients diagnosed with AOID, 58 experienced pustular reactions. From these 58 individuals, seventeen blood samples were specifically collected for genetic analysis. Additionally, in the group of sixty-seven identified cases of PP, 24 blood samples were obtained. Consequently, the genetic study encompassed a total of 41 patients. The recruitment and selection process for these patients is detailed in the study flow diagram presented in Figure 2A.

Among 17 patients diagnosed with AOID with pustular reaction, the mean age of onset was 52.9 ± 12.0 years. Nine (52.9%) were male. Regarding the living area, 58.8% of them had been living in northeastern Thailand, while 41.2% had been living in northern Thailand.

The mean age of onset was 40.4 ± 24.0 years among 24 PP cases, and 6 of them (25%) were male. The majority (62.5%) had been living in northern Thailand, while the rest of them (37.5%) had been living in northeastern Thailand. Generalized pustular psoriasis (GPP) was the most prevalent subtype, followed by acrodermatitis continua of Hallopeau and localized pustular psoriasis (75%, 16.7%, and 8.3%, respectively).

### 3.2. HLA Types Are Associated with Pustular Skin Diseases

HLA typing of 41 patients with pustular skin diseases was carried out based on next-generation sequencing (NGS) technology. The frequencies of HLA class I (A, B, and C) and class II (DPB1, DQB1, and DRB1) alleles identified in this study are summarized in Supplementary Table S1. In our cohort, HLA-A*11:01 (AF = 0.280), HLA-B*13:01 (AF = 0.195), and HLA-C*03:04 (AF = 0.244) were mostly observed for HLA class I, and HLA-DQB1*05:02 (AF = 0.366), DRB1*15:02 (AF = 0.232), and DPB1*05:01 (AF = 0.305) were mostly observed for HLA class II. Interestingly, patients who carry at least one of the DQB1*05:01 or DQB1*05:02 alleles account for 76% of the dataset. Moreover, 73% of the patients in this cohort carry at least one DRB1*15:01 or DRB1*15:02 allele and one DPB1*05:01 or DPB1*13:01 allele.

The association between the patients who carry pustular skin diseases and HLA alleles was evaluated by comparing them with the HLA allele frequency of the Thai healthy population (N = 470) randomly chosen from five regional groups of Thailand reported in Satapornpong et al., 2020 [21]. We observed that seven HLA alleles, three from HLA class I and four alleles from class II, were significantly associated with pustular skin diseases, i.e., HLA-B*13:01 (OR = 3.825, 95%CI: 2.08–7.035), B*18:02 (OR = 3.154, 95%CI: 1.002–9.73), C*03:04 (OR = 3.665, 95%CI: 2.102–6.39), DQB1*03:01 (OR = 0.448, 95%CI: 0.203–0.991), DQB1*05:02 (OR = 2.134, 95%CI: 1.326–3.434), DRB1*15:01 (OR = 2.971, 95%CI: 1.658–5.324), and DRB1*15:02 (OR = 1.783, 95%CI: 1.034–3.072) (Table 1). To confirm these associations, we applied different control groups and observed the consensus. The HLA allele frequency of the Thai healthy population (N = 334) from a dengue vaccine efficacy trial conducted in Ratchaburi Province in Thailand and reported by Geretz et al., 2018 [20], was used as a control group for validation. We found that six of the seven HLA alleles remained significantly associated with pustular skin diseases, i.e., HLA-B*13:01 (OR = 3.909, 95%CI: 2.072–7.274), C*03:04 (OR = 3.745, 95%CI: 2.103–6.668), DQB1*03:01 (OR = 0.398, 95%CI: 0.179–0.884), DQB1*05:02 (OR = 2.557, 95%CI: 1.566–4.174), DRB1*15:01 (OR = 2.805, 95%CI: 1.541- 5.105), and DRB1*15:02 (OR = 2.576, 95%CI: 1.457–4.554) (Supplementary Table S2). There were additional significantly associated alleles when the latter was used as a control group, i.e., HLA-A*33:03 (OR = 0.338, 95%CI: 0.121–0.947), A*13:01 (OR = 0.391, 95%CI: 2.072–7.374), DPB1*05:01 (OR = 3.746, 95%CI: 2.202–6.374), DQB1*05:01 (OR = 2.233, 95%CI: 1.158–4.307), and DRB1*16:02 (OR = 2.23, 95%CI: 1.031–4.823). However, it is important to note that the *HLA-DPB1* gene was not present in the first comparison because it was not included by the original study. Therefore, we included the *DPB1* gene in the overlapped list, which was B*13:01, C*03:04, DPB1*05:01, DQB1*03:01, DQB1*05:02, DRB1*15:01, and DRB1*15:02. These seven alleles were associated with the patients who developed sterile skin pustules consistently from both control datasets. Of these, six alleles were observed to increase the risk of pustular skin reaction (OR > 1), and only DQB1*03:01 was observed to have a protective role against the reaction (OR < 1).

We further evaluated the association of these alleles in each disease group of the pustular diseases. Of those seven alleles, the association of three’ alleles remained significant in both separated conditions of AOID vs. healthy and PP vs. healthy, i.e., B*13:01, C*03:04, and DQB1*05:02 (Supplementary Tables S3 and S4), while the association of DQB1*03:01 disappeared from both conditions (Figure 2B). Interestingly, the two alleles DPB1*05:01 (OR = 3.65, 95%CI: 1.57–8.46) and DRB1*15:02 (OR = 4.66, 95%CI: 2.21–9.82) were significantly associated with pustular reaction in only the AOID group, while DRB1*15:01 (OR = 4.476, 95%CI: 2.26–8.86) was significantly associated with only pustular reaction in the PP group.

### 3.3. Enrichment Analysis of HLA Variants in the Pustular Skin Diseases

We further performed a variant enrichment analysis to determine whether a particular variant is consistently abundant in the patient group in comparison to the general population. Here, the frequency of variants, including single nucleotide variants (SNVs), insertion/deletion (INDEL), and frameshift variants found in the HLA candidate genes (*HLA-B*, *C*, and *DQB1*), was compared to that of the East Asian population (EAS) consisting of Han Chinese, Japanese, Southern Han Chinese, Chinese Dai, and Kinh populations from a catalog of common human genetic variation (1000 genome project database) [23] and the Genome Aggregation Database (gnomAD) [24]. From a total of 216 missense and 23 frameshift variants identified in these three HLA genes, 52 missense and 8 frameshift variants were observed to be significantly enriched in the subjects presented with pustular skin diseases (Supplementary Table S5).

**Figure 3:**
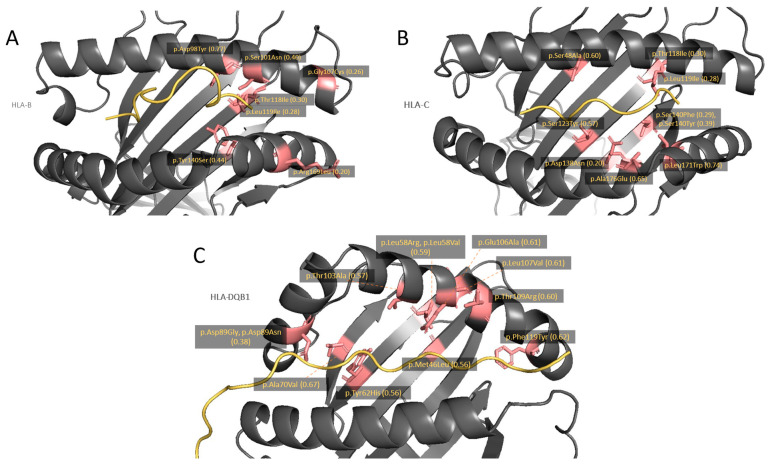
Detected missense SNVs in the peptide-binding groove of *HLA-B* (**A**), *HLA-C* (**B**), and *HLA-DQB1* (**C**) molecules. HLA molecules are shown in a black ribbon, while peptide antigens are shown in the yellow line. The locations of amino acid change according to the missense SNVs, which are significantly enriched in pustular skin disease dataset, are emphasized by salmon pink color. Text labels show amino acid changes and their allele frequency (*n* = 82).

To further investigate the potential effect of these variants on HLA protein function, we reconstructed HLA protein structures, mapped the variants, accordingly, and studied them in the context of the annotated HLA functional domains (Supplementary Table S5). Interestingly, many of those variants were identified in the antigen-binding groove (11 out of 23 variants in *HLA-B*, 9 out of 17 variants in *HLA-C*, 12 out of 20 variants in *HLA-DQB1*) (Figure 3 and Supplementary Table S5). Of note, a primary activity of HLA is to load endogenous and exogenous peptides into the binding groove and present them to immune cells. Therefore, variants leading to amino acid change in the binding groove are likely to impact the HLA function and, thus, the regulation of immune response.

**Figure 4:**
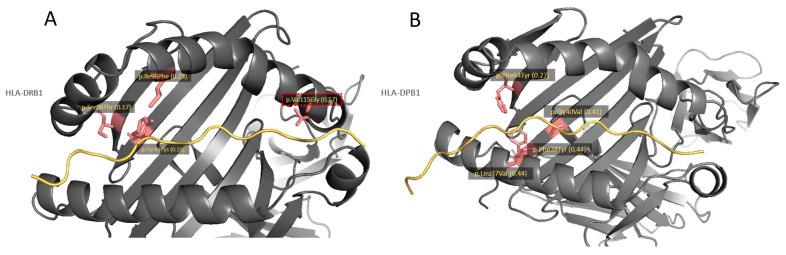
Detected missense SNVs in the peptide-binding groove of *HLA-DRB1* (**A**) and *HLA-DPB1* (**B**) molecules. HLA molecules are shown in a black ribbon, while peptide antigens are shown in the yellow line. The locations of amino acid change according to the missense SNVs, which are significantly enriched in pustular skin disease dataset, are emphasized by salmon pink color. Text labels show amino acid changes and their allele frequency (*n* = 82). HLA variant associated with pustular reaction in AOID by using PP as a control is emphasized by red dash box.

We further identified the variants that were enriched in *DPB1* and *DRB1* genes as they can potentially lead to separate entities of AOID and PP. It was found that 13 missense (5 in *DPB1* and 8 in *DRB1*) and 4 frameshift variants (1 in *DPB1* and 3 in *DRB1*) out of 90 missense and 21 frameshift variants were observed to be significantly enriched in *DPB1* and *DRB1* genes in the subjects presented with pustular skin diseases (Supplementary Table S5). Among these, four missense variants of *DPB1* (Figure 4A) and four missense and three frameshift variants of *DRB1* were localized in the binding groove (Figure 4B).

### 3.4. HLA Alleles Can Be Used to Distinguish Pustular Reaction in AOID from PP

It has been well evidenced that pustular reaction in AOID shares a similar clinical phenotype with PP. However, the biomarker for separating the two conditions has not been reported. Therefore, we performed further analysis to identify the potential HLA alleles that could be used to distinguish between pustular reactions in AOID and PP patients. Using PP patients as a control, the odd ratio of HLA alleles in pustular reaction in AOID patients showed that DPB1*05:01 (OR = 3.851, 95%CI: 1.432–10.36, *p* = 0.008) and DRB1*15:02 (OR = 3.195, 95%CI: 1.1–9.281, *p* = 0.033) have a significant association with pustular reaction in AOID patients (Supplementary Table S6). This, together with the previous result, indicates that DPB1*05:01 and DRB1*15:02 alleles are a risk factor for sterile skin pustules in AOID patients.

We further identified HLA variants associated with pustular reaction in AOID by using PP as a control. From all 77 variant candidates enriched in *HLA-B*, *C*, *DQB1*, *DPB1*, and *DRB1* genes in our dataset, we identified 4 SNVs, rs9269744 (OR = 0.048, *p* = 0.020), rs77637983 (OR = 0.035, *p* = 0.009), rs17885482 (OR = 0.110, *p* = 0.005), and rs9270302 (OR = 0.035, *p* = 0.009), associated with pustular reaction in AOID by logistic regression adjusted for age and gender. The less stringent multiple-comparison correction method based on empirical distribution confirmed these associations (Table 2), despite the conservative Bonferroni method being unable to preserve these signals. Interestingly, all of these variants are in the *DRB1* gene suggesting an important role of this gene in pustular reaction formation in AOID patients. By using PP patients as the case and AOID patients as the control group, the odd ratios of rs9269744, rs77637983, rs17885482, and rs9270302 were 21.04, 28.96, 9.073, and 28.96, respectively. Linkage disequilibrium (LD) analysis based on the *r^2^* metric showed that three SNVs, rs77637983, rs9270302, and rs9269744, are tightly linked (*r^2^ > 0.8*), and only rs17885482 is independent from the others (Supplementary Table S7).

To confirm whether these variants are from the *DRB1* alleles, we examined the association between the presence of alleles, i.e., HLA-DRB1*15:01 and HLA-DRB1*15:02, and the presence of those variants. Only rs17885482 was observed to be associated with DRB1*15:01 (OR = 25.93, *p* = 0.002) and DRB1*15:02 (OR = 0.158, *p* = 0.006), confirming that rs17885482 belongs to the DRB1*15:01 allele (Supplementary Table S8). However, we could not observe an association between the *DRB1* alleles and the tight LD of SNVs (rs77637983, rs9270302, and rs9269744), suggesting that these variants are independent of the HLA allele.

## 4. Discussion

The pathogenesis of pustular skin diseases is multifactorial and remains largely unelucidated [26]. While genetic factors such as mutations in IL36RN, CARD14, and AP1S3 are proposed to enhance the inflammatory cascade in several cellular subtypes, the high genetic heterogeneity of these diseases leads to ambiguous conclusions. The manifestation of sterile pustules in PP, similar to AOID patients, provides a crucial clue for investigating common genetic factors that increase the risk of this phenotype.

The association between HLA genes and immune-mediated diseases, including PP [27] and AOID [16], has been well documented. Therefore, we hypothesized that the HLA genes also play a role in pustule formation. In this study, specific HLA alleles were reported to be significantly associated with the presence of sterile skin pustules in pustular skin patients compared with the non-disease population, suggesting that HLA polymorphism is one of the genetic factors of the key etiopathogenesis in these pustular skin diseases.

Specifically, in this study, we found that HLA-B*13:01, HLA-C*03:04, and DQB1*05:02 are shared in PP and pustular reaction in AOID, implying that, up to a certain point, there might be a common pathway contributing to pustule formation (Figure 2B). A thorough literature review concluded that there is no evidence of these HLA alleles in connection with any pustular diseases. Therefore, this is the first report suggesting the possible association of the aforementioned HLA alleles as a risk factor for pustular skin reaction. Interestingly, the HLA-B*13:01 allele has been shown to strongly correlate with hypersensitivity reactions to dapsone [28] and severe cutaneous adverse drug reactions to dapsone in the Thai population. As dapsone is considered as an alternative treatment for PP [29], caution should be used in prescribing dapsone in patients with PP.

As was to be expected, there was also some divergence of *HLA-DPB1* and *DRB1* alleles between PP and pustular reaction in AOID, supporting the fact that they are separate entities evidenced by their distinctive extracutaneous features and other systemic manifestations. Notably, HLA-DPB1*05:01 showed significant association only with AOID patients with pustular skin reaction. Several studies have consistently shown that the HLA-DPB1*05:01 allele is associated with an increased risk of various autoimmune diseases, such as rheumatoid arthritis, systemic lupus erythematosus, aplastic anemia, and systemic sclerosis, particularly within the Asian population [30,31,32]. This underscores the significant role played by this allele in autoimmune conditions. Here, we report the first evidence of the HLA-DPB1*05:01 allele in association with the occurrence of pustular skin reaction in AOID patients, representing a novel biomarker to distinguish pustular skin reactions in AOID from PP patients. The DRB1*15:01 [16] and DRB1*15:02 [33] alleles have been previously reported to be related to AOID. Interestingly, in our study, the DRB1*15:01 allele was related to pustular reaction in PP patients, whereas the DRB1*15:02 allele was related to pustular reaction in AOID patients using healthy individuals as the control group. Our subsequent investigation comparing patients with pustular reaction in AOID to those with pustular reaction in PP revealed significant results confirming this signal in the DRB1*15:02 allele. The amino acid sequence alignment of the DRB1*15:01 and the DRB1*15:02 alleles revealed that both sequences are nearly identical, with only one amino acid difference at position 115 (p.Val115Gly) (Supplementary Figure S1). Therefore, further investigation on this variant is required to determine its impact on the antigen presentation process and pathogenesis of the two diseases.

Interestingly, a number of HLA class II missense variants, i.e., *HLA-DPB1*, *DRB1*, and *DQB1*, that are enriched in this dataset are more in the antigen-binding groove (67%, 64%, and 60%, respectively) compared to class I, i.e., *HLA-B* (48%) and *HLA-C* (53%). This high variation in the binding groove of HLA class II may play an important role in the disease pathogenesis by altering regulation or lowering the selection threshold for autoreactive T cells during T-cell development in the thymus [34,35]. Among these variants, we again identified rs17885482 (p.Val115Gly) in the *HLA-DRB1* gene as a biomarker for separating pustular skin reactions in AOID and PP patients. Numerous studies have shown that the variants on the HLA-DR binding groove could directly impact the binding affinity of exogenous antigens in presenting to CD4+ T cells and relate to autoimmune disease [34,36,37]. Therefore, particular environmental triggers might be involved in predisposing individuals to pustular skin diseases.

The therapeutic implications of our study on both AOID and PP are extensive and multifaceted, especially in the context of early diagnosis and tailored interventions. Our insights into specific HLA polymorphisms serve as an important element in enhancing diagnostic accuracy enabling early identification of individuals presenting with multiple sterile skin pustules without known documented underlying conditions. This early detection, facilitated by the identified specific HLA polymorphisms, offers a potential for timely and effective therapeutic interventions, which is critical given the nature of AOID and PP where a delay in diagnosis can lead to advanced disease progression and increased morbidity. Our research may contribute to understanding factors that could potentially reduce the mortality rate associated with these diseases by allowing for the early identification of at-risk individuals and the development of targeted treatment protocols.

Interestingly, nearly all AOID patients reported to date originate from Asia or have Asian ancestry, even if they reside elsewhere [4]. This suggests a notable racial predisposition within the population. Our study contributes significantly to the understanding of AOID’s prevalence and characteristics, especially in East and Southeast Asian populations. It highlights the influence of specific infections or environmental factors unique to these regions and underscores the pronounced linkage of the disease to the Japanese population. This geographical and ethnic specificity is vital for clinicians to consider in the clinical interpretation of AOID and similar conditions, as it provides essential insights into the disease’s etiology and the tailoring of treatment strategies. However, it is important to note the limitation in our findings’ applicability to Western or African populations, given the almost exclusive Thai background of our patient cohort. This highlights the need for further research involving diverse demographic settings to validate and expand upon our findings, ensuring global applicability and relevance.

Additionally, the genetic insights garnered from our research could inform future clinical trials by identifying subsets of patients who are more likely to respond to specific treatments. This would not only enhance the efficacy and efficiency of such trials but also contribute to a more nuanced understanding of patient responsiveness to various therapeutic interventions. The knowledge gained from our study is instrumental in counseling patients and their families about the disease prognosis and management strategies, thereby contributing to a more comprehensive and holistic approach to patient care.

A limitation of this study is the relatively small number of patients due to the rarity of the diseases. Also, a healthy control cohort collected in parallel with the case collection should be applied to avoid the confounding effect of geographic location. While the small patient sample is a limitation due to the rare nature of AOID, it is also a strength of this study that we were able to collect a substantial number of samples within this context. The 17 blood samples from AOID patients with pustular reactions, though limited, represent a significant achievement in studying such a rare condition. Coupled with the potential geographic bias and the absence of a control group, these limitations are balanced by the unique opportunity this study had in gathering data on a rare disease. The inclusion of acute generalized exanthematous pustulosis (AGEP) was constrained due to diagnostic practices, yet the HLA polymorphisms identified, particularly HLA-DPB1*05:01 and DRB1*15:02 alleles and the rs17885482 variant in DRB1*15:01, emerge as promising biomarkers for differentiating pustular skin diseases in AOID and PP patients. An additional limitation of this study is its potential lack of applicability to populations outside of Thailand. Given that our patient cohort predominantly comprises Thai individuals, the results may not be directly transferable to Western or African populations. This geographic and ethnic specificity should be considered when interpreting our findings, especially in the context of global applicability. Further studies involving diverse populations are necessary to validate and extend our results to a broader demographic spectrum.

In expanding our investigation into the genetic underpinnings of pustular skin diseases, recent literature and whole-exome sequencing (WES) analyses have unveiled significant genetic variations beyond HLA polymorphisms. Notably, variations in six genes, IL1RN, IL36RN, CARD14, AP1S3, MPO, and TNIP1 [1], have been implicated in the pathophysiology of these conditions, enhancing the inflammatory response through the activation of IL-1 and IL-36 cytokine signaling pathways. This is particularly evident in dendritic cells, where IL-36 activation promotes the expression of HLA class II and co-stimulatory molecules CD80 and CD86 [38]. However, the high genetic heterogeneity of these diseases complicates the delineation of clear genetic risk variants, with known variants explaining only approximately one-third of the total case number [39]. This underscores the necessity for elucidating additional genetic predisposing factors. Recent WES analyses of three unrelated Thai patients revealed rare genetic variants within the SERPINB3 gene [40], linking GPP and AOID through common pathogenetic pathways and supporting the hypothesis of a shared genetic foundation.

In conclusion, our study enriches the genetic discourse on pustular skin diseases by identifying HLA polymorphisms as potential biomarkers. However, the recognition of additional genetic variations emphasizes the complexity of these diseases’ genetic architecture. Our findings lay the groundwork for future research, calling for a comprehensive approach to unravel the genetic predispositions of pustular skin diseases fully. This holistic exploration is crucial for developing targeted diagnostic and therapeutic strategies, thereby enhancing patient care and outcomes in the face of these challenging conditions.

## Figures and Tables

**Table 1:** Odd ratio of HLA alleles in 41 patients with pustular skin diseases in comparison with Thai healthy controls (Satapornpong et al., 2020) [21].

Allele	2n = 82	AF (2n = 82)	AF Thai Healthy (2n = 940)	2n = 940	Odds Ratio	95% CI	*p*-Value
A*33:03	4	0.049	0.1117	105	0.408	0.146 to 1.137	0.086
B*13:01	16	0.195	0.0596	56	3.825	2.08 to 7.035	**<0.001**
B*18:02	4	0.049	0.016	15	3.154	1.022 to 9.73	**0.046**
C*03:04	20	0.244	0.081	76	3.665	2.102 to 6.39	**<0.001**
DPB1*05:01 *	25	0.305	NA	NA	NA	NA	NA
DQB1*03:01	7	0.085	0.1723	162	0.448	0.203 to 0.991	**0.047**
DQB1*05:01	13	0.159	0.1404	132	1.154	0.62 to 2.145	0.652
DQB1*05:02	30	0.366	0.2128	200	2.134	1.326 to 3.434	**0.002**
DRB1*15:01	17	0.207	0.081	76	2.971	1.658 to 5.324	**<0.001**
DRB1*15:02	19	0.232	0.145	136	1.783	1.034 to 3.072	**0.037**
DRB1*16:02	9	0.11	0.0596	56	1.945	0.925 to 4.09	0.079

Abbreviation: AF = allele frequency; CI = confidence interval; NA = not available. Alleles with significant association (*p*-Value < 0.05) to pustular skin diseases were highlighted in bold. * DPB1 allele frequency is not available in [21], but available in [20].

**Table 2:** Association between HLA variants and pustular reaction in AOID using PP patients as control.

Location	Alt	Accession ID	Binding Pocket	Odds	P	Emp P
6:32580249 -32580249	C	rs9269744	N	0.048	0.020	0.004
6:32581675 -32581675	G	rs77637983	N	0.035	0.009	0.001
6:32584135 -32584135	C	rs17885482	Y	0.110	0.005	<0.001
6:32589702 -32589702	A	rs9270302	N	0.035	0.009	0.001

Abbreviation: Alt, alternative allele; AOID, adult-onset immunodeficiency; Odd, odd ratio; P, *p*-value; Enriched, significantly enriched in pustular skin disease dataset: Emp P: empirical *p*-value based on empirical distribution of 10,000 permutations.

## Data Availability

The raw genetic data used in the current study are available from the corresponding author on reasonable request. All code and data generated or analyzed during this study are deposited at https://data.mendeley.com/datasets/cbvw68rd9k (accessed on 20 February 2024).

## Supplementary Materials

The following supporting information can be downloaded at https://www.mdpi.com/article/10.3390/genes15030278/s1, Figure S1: Sequence alignment result between *DRB1*15:01* and *DRB1*15:02* using EMBOSS Needle method; Table S1: HLA frequency in 41 patients with pustular skin diseases; Table S2: Odd ratio of HLA alleles in 41 pustular skin diseases in comparison with Thai healthy controls (dengue vaccine efficacy trial cohort) [5]; Table S3: Odd ratio of HLA alleles in 17 pustular reactions in AOID patients in comparison with Thai healthy controls; Table S4: Odd ratio of HLA alleles in 24 pustular psoriasis patients in comparison with Thai healthy controls; Table S5: Enriched mutations that cause amino acid change in HLA genes in patients with pustular skin diseases; Table S6: Odd ratio of HLA alleles in pustular reaction in AOID patients in comparison with pustular psoriasis patient control; Table S7: Linkage disequilibrium analysis based on r2 metric of AOID-associated SNVs; Table S8: Association between HLA variants and DRB1 alleles.
