# Supplementary material for: Human Leukocyte Antigen Markers for Distinguishing Pustular Psoriasis and Adult-Onset Immunodeficiency with Pustular Reaction
Source: Genes (Basel). 2024 Feb 23;15(3):278. doi: 10.3390/genes15030278 (PMC10970016; doi:10.3390/genes15030278)
Supplement: Supplementary file 1 [file genes-15-00278-s001.zip › Figure S1.pdf]

Figure S1 Sequence alignment result between *DRB1*15:01* and *DRB1*15:02* using EMBOSS Needle method

```
01                  1 MVCLKLPGGSCMTALTVTLMVLSSPLALSGDTRPRFLWQPKRECHFFNGT     50
                      ||||||||||||||||||||||||||||||||||||||||||||||||||
02                  1 MVCLKLPGGSCMTALTVTLMVLSSPLALSGDTRPRFLWQPKRECHFFNGT     50

01                 51 ERVRFLDRYFYNQEESVRFDSDVGEFRAVTELGRPDAEYWNSQKDILEQA    100
                      ||||||||||||||||||||||||||||||||||||||||||||||||||
02                 51 ERVRFLDRYFYNQEESVRFDSDVGEFRAVTELGRPDAEYWNSQKDILEQA    100

01                101 RAAVDTYCRHNYGVVESFTVQRRVQPKVTVYPSKTQPLQHHNLLVCSVSG    150
                      ||||||||||||||.|||||||||||||||||||||||||||||||||||
02                101 RAAVDTYCRHNYGVGESFTVQRRVQPKVTVYPSKTQPLQHHNLLVCSVSG    150

01                151 FYPGSIEVRWFLNGQEEKAGMVSTGLIQNGDWTFQTLVMLETVPRSGEVY    200
                      ||||||||||||||||||||||||||||||||||||||||||||||||||
02                151 FYPGSIEVRWFLNGQEEKAGMVSTGLIQNGDWTFQTLVMLETVPRSGEVY    200

01                201 TCQVEHPSVTSPLTVEWRARSESAQSKMLSGVGGFVLGLLFLGAGLFIYF    250
                      ||||||||||||||||||||||||||||||||||||||||||||||||||
02                201 TCQVEHPSVTSPLTVEWRARSESAQSKMLSGVGGFVLGLLFLGAGLFIYF    250

01                251 RNQKGHSGLQPTGFLS    266
                      ||||||||||||||||
02                251 RNQKGHSGLQPTGFLS    266
```

01 = DRB1*15:01:01:01
02 = DRB1*15:02:01:02
# Matrix: EBLOSUM62
# Gap_penalty: 10.0
# Extend_penalty: 0.5
#
Length: 266
Identity: 265/266 (99.6%)
Similarity: 265/266 (99.6%)
Gaps: 0/266 (0.0%)
Score: 1404.0

HLA allele sequences were retrieved from https://www.ebi.ac.uk/ipd/imgt/hla
Pairwise Sequence alignment was performed using https://www.ebi.ac.uk/Tools/psa/emboss_needle/
