# Supplementary material for: Human Leukocyte Antigen Markers for Distinguishing Pustular Psoriasis and Adult-Onset Immunodeficiency with Pustular Reaction
Source: Genes (Basel). 2024 Feb 23;15(3):278. doi: 10.3390/genes15030278 (PMC10970016; doi:10.3390/genes15030278)
Supplement: Supplementary file 1 [file genes-15-00278-s001.zip › TableS1.pdf]

Table S1 HLA frequency in 41 patients with pustular skin diseases

A	count	AF	B	Count	AF	C	Count	AF	DPB1	Count	AF	DQB1	Count	AF	DRB1	Count	AF
**02:03**	12	0.146	**13:01**	16	0.195	**01:02**	11	0.134	**02:01**	13	0.159	**03:01**	7	0.085	**04:05**	3	0.037
**02:06**	3	0.037	**15:02**	5	0.061	**03:04**	20	0.244	**02:02**	5	0.061	**03:03**	13	0.159	**09:01**	12	0.146
**02:07**	11	0.134	**15:25**	4	0.049	**04:03**	4	0.049	**03:01**	3	0.037	**05:01**	13	0.159	**12:02**	7	0.085
**11:01**	23	0.28	**18:01**	3	0.037	**07:02**	11	0.134	**05:01**	25	0.305	**05:02**	30	0.366	**14:04**	3	0.037
**11:02**	4	0.049	**18:02**	4	0.049	**07:04**	6	0.073	**13:01**	18	0.22	**05:03**	3	0.037	**15:01**	17	0.207
**24:02:00**	9	0.11	**38:02:00**	3	0.037	**08:01**	8	0.098	**14:01**	5	0.061	**06:01**	7	0.085	**15:02**	19	0.232
**24:07:00**	6	0.073	**40:01:00**	7	0.085	**14:02**	5	0.061	**21:01**	3	0.037				**16:02**	9	0.11
**24:10:00**	4	0.049	**46:01:00**	13	0.159												
**33:03:00**	4	0.049	**51:01:00**	5	0.061												

AF; allele frequency
