# Supplementary material for: Human Leukocyte Antigen Markers for Distinguishing Pustular Psoriasis and Adult-Onset Immunodeficiency with Pustular Reaction
Source: Genes (Basel). 2024 Feb 23;15(3):278. doi: 10.3390/genes15030278 (PMC10970016; doi:10.3390/genes15030278)
Supplement: Supplementary file 1 [file genes-15-00278-s001.zip › TableS2.pdf]

Table S2 Odd ratio of HLA alleles in 41 pustular skin diseases in comparison with Thai healthy controls (dengue vaccine efficacy trial cohort) [5]

HLA allele	2n=82	AF (2n=82)	AF Thai Healthy (2n=668)	2n=668	Odds ratio	95% CI	P-value
A*33:03	4	0.049	0.132	88	0.338	0.121 to 0.947	0.039
B*13:01	16	0.195	0.058	39	3.909	2.072 to 7.374	<0.001
B*18:02	4	0.049	0.025	17	1.968	0.646 to 5.997	0.234
C*03:04	20	0.244	0.079	53	3.745	2.103 to 6.668	<0.001
DPB1*05:01	25	0.305	0.105	70	3.746	2.202 to 6.374	<0.001
DQB1*03:01	7	0.085	0.19	127	0.398	0.179 to 0.884	0.024
DQB1*05:01	13	0.159	0.078	52	2.233	1.158 to 4.307	0.016
DQB1*05:02	30	0.366	0.184	123	2.557	1.566 to 4.174	<0.001
DRB1*15:01	17	0.207	0.085	57	2.805	1.541 to 5.105	0.001
DRB1*15:02	19	0.232	0.105	70	2.576	1.457 to 4.554	0.001
DRB1*16:02	9	0.11	0.052	35	2.23	1.031 to 4.823	0.042
