# Supplementary material for: Human Leukocyte Antigen Markers for Distinguishing Pustular Psoriasis and Adult-Onset Immunodeficiency with Pustular Reaction
Source: Genes (Basel). 2024 Feb 23;15(3):278. doi: 10.3390/genes15030278 (PMC10970016; doi:10.3390/genes15030278)
Supplement: Supplementary file 1 [file genes-15-00278-s001.zip › TableS3.pdf]

Table S3 Odd ratio of HLA alleles in 17 pustular reactions in AOID patients in comparison with Thai healthy controls

AOID patients			VS Thai healthy controls (Satapornpong et al., 2020)					VS Thai healthy controls (Geretz et al., 2018)				
HLA type	AF (2n=46)	2n	AF (2n=940)	2n	Odds	P-value	CI 95%	AF (2n=668)	2n	Odds	P-value	CI 95%
A*02:03	0.235	8	0.112	105	2.447	0.0320	1.08 to 5.545	0.0943	63	2.955	0.0108	1.284 to 6.803
A*11:01	0.294	10	0.261	245	1.182	0.663	0.557 to 2.508	0.2485	166	1.260	0.549	0.59 to 2.69
A*11:02	0.088	3	0.018	17	5.250	0.0110	1.462 to 18.852	0.0329	22	2.845	0.103	0.808 to 10.019
A*24:02	0.118	4	0.115	108	1.027	0.961	0.355 to 2.972	0.1228	82	0.952	0.929	0.327 to 2.773
A*24:07	0.088	3	0.043	40	0.94	0.214	0.638 to 7.416	0.0524	35	1.750	0.373	0.51 to 6.005
B*13:01	0.206	7	0.060	56	4.091	0.00158	1.707 to 9.804	0.0584	39	4.180	0.0016	1.713 to 10.199
B*40:01	0.147	5	0.066	62	2.440	0.0754	0.913 to 6.523	0.0853	57	1.849	0.222	0.689 to 4.962
B*46:01	0.88	3	0.140	132	0.593	0.392	0.179 to 1.966	0.1123	75	0.765	0.664	0.228 to 2.563
C*03:04	0.324	11	0.081	76	5.433	<0.0001	2.552 to 11.57	0.079300	53	5.553	<0.0001	2.567 to 12.009
C*07:02	0.176	6	0.119	112	1.585	0.318	0.642 to 3.912	0.1497	100	1.217	0.671	0.491 to 3.015
C*07:04	0.088	3	0.05	47	1.839	0.328	0.542 to 6.233	0.0419	28	2.213	0.211	0.638 to 7.678
C*08:01	0.088	3	0.103	97	0.841	0.778	0.252 to 2.802	0.1078	72	0.801	0.719	0.239 to 2.686
DPB1*02:01	0.088	3	NA	NA	NA	NA	NA	0.1003	67	0.868	0.819	0.258 to 2.916
DPB1*05:01	0.471	16	NA	NA	NA	NA	NA	0.1048	70	7.593	<0.0001	3.705 to 15.56
DPB1*13:01	0.176	6	NA	NA	NA	NA	NA	0.1751	117	1.010	0.984	0.409 to 2.493
DQB1*03:03	0.118	4	0.113	106	1.049	0.930	0.362 to 3.035	0.1228	82	0.952	0.929	0.327 to 2.773
DQB1*05:01	0.235	8	0.140	132	1.884	0.127	0.835 to 4.25	0.077800	52	3.647	0.0254	1.572 to 8.461
DQB1*05:02	0.382	13	0.213	200	2.29	0.0200	1.127 to 4.654	0.1841	123	2.744	0.00581	1.337 to 5.63
DQB1*06:01	0.088	3	0.071	67	1.261	0.708	0.376 to 4.231	0.0928	62	0.946	0.929	0.281 to 3.184
DRB1*04:05	0.088	3	0.049	46	1.882	0.310	0.555 to 6.386	0.0539	36	1.699	0.399	0.496 to 5.822
DRB1*09:01	0.118	4	0.099	93	1.215	0.720	0.419 to 3.524	0.1078	72	1.104	0.857	0.378 to 3.222
DRB1*15:01	0.118	4	0.081	76	1.515	0.447	0.52 to 4.413	0.0853	57	1.43	0.515	0.487 to 4.202
DRB1*15:02	0.353	12	0.145	136	3.224	0.00159	1.559 to 6.667	0.1048	70	4.659	<0.0001	2.21 to 9.821
DRB1*16:02	0.265	9	0.060	56	5.680	<0.0001	2.531 to 12.748	0.0524	35	6.510	<0.0001	2.826 to 14.997

*Yellow highlight indicates a statistically significant association
