# Supplementary material for: Human Leukocyte Antigen Markers for Distinguishing Pustular Psoriasis and Adult-Onset Immunodeficiency with Pustular Reaction
Source: Genes (Basel). 2024 Feb 23;15(3):278. doi: 10.3390/genes15030278 (PMC10970016; doi:10.3390/genes15030278)
Supplement: Supplementary file 1 [file genes-15-00278-s001.zip › TableS4.pdf]

Table S4 Odd ratio of HLA alleles in 24 pustular psoriasis patients in comparison with Thai healthy controls

PP patients			VS Thai healthy controls (Sataspornpong et al., 2020)					VS Thai healthy controls (Geretz et al., 2018)				
HLA type	AF (2n=46)	2n	AF (2n=940)	2n	Odds	P-value	CI 95%	AF (2n=668)	2n	Odds	P-value	CI 95%
A*02:03	0.087	4	0.1117	105	0.757	0.602	0.266 to 2.155	0.094	63	0.915	0.869	0.318 to 2.635
A*02:07	0.197	9	0.084	79	2.653	0.012	1.235 to 5.695	0.088	59	2.511	0.020	1.156 to 5.457
A*11:01	0.283	13	0.261	245	1.118	0.740	0.579 to 2.159	0.249	166	1.191	0.606	0.612 to 2.317
A*24:02	0.109	5	0.1149	108	0.939	0.897	0.363 to 2.429	0.123	82	0.871	0.777	0.335 to 2.268
A*24:07	0.065	3	0.0426	40	1.568	0.467	0.466 to 5.271	0.052	35	1.262	0.709	0.373 to 4.269
A*24:10	0.065	3	0.017	16	4.034	0.031	1.132 to 14.374	0.020	13	3.508	0.057	0.963 to 12.776
A*33:03	0.087	4	0.1117	105	0.757	0.602	0.266 to 2.155	0.132	88	0.628	0.385	0.22 to 1.794
B*13:01	0.196	9	0.0596	56	3.838	0.001	1.765 to 8.347	0.058	39	3.922	0.001	1.767 to 8.702
B*15:02	0.065	3	0.0766	72	0.841	0.776	0.255 to 2.778	0.091	61	0.694	0.551	0.209 to 2.305
B*15:25	0.065	3	0.0223	21	3.059	0.079	0.878 to 10.654	0.024	16	2.837	0.108	0.796 to 10.113
B*46:01	0.217	10	0.1404	132	1.701	0.151	0.824 to 3.509	0.112	75	2.196	0.037	1.047 to 4.605
B*51:01	0.087	4	0.0426	40	2.140	0.165	0.732 to 6.261	0.039	26	2.353	0.127	0.785 to 7.055
C*01:02	0.217	10	0.1713	161	1.344	0.422	0.654 to 2.763	0.139	93	1.718	0.149	0.824 to 3.579
C*03:04	0.196	9	0.0809	76	2.763	0.009	1.286 to 5.94	0.079	53	2.824	0.009	1.294 to 6.165
C*04:03	0.065	3	0.0426	40	1.568	0.467	0.466 to 5.271	0.054	36	1.225	0.744	0.362 to 4.138
C*07:02	0.109	5	0.1191	112	0.902	0.831	0.349 to 2.33	0.150	100	0.693	0.450	0.267 to 1.796
C*07:04	0.065	3	0.05	47	1.326	0.647	0.397 to 4.43	0.042	28	1.595	0.457	0.466 to 5.458
C*08:01	0.109	5	0.1032	97	1.060	0.905	0.409 to 2.745	0.108	72	1.009	0.985	0.386 to 2.636
C*14:02	0.087	4	0.0287	27	3.223	0.036	1.079 to 9.632	0.034	23	2.673	0.082	0.884 to 8.086
DPB1*02:01	0.217	10	NA	NA	NA	NA	NA	0.100	67	2.492	0.016	1.183 to 5.247
DPB1*02:02	0.065	3	NA	NA	NA	NA	NA	0.067	45	0.965	0.954	0.288 to 3.234
DPB1*05:01	0.196	9	NA	NA	NA	NA	NA	0.213	142	0.901	0.786	0.425 to 1.91
DPB1*13:01	0.261	12	NA	NA	NA	NA	NA	0.175	117	1.663	0.147	0.836 to 3.307
DPB1*14:01	0.087	4	NA	NA	NA	NA	NA	0.028	19	3.258	0.039	1.06 to 10.012
DQB1*03:01	0.130	6	0.1723	162	0.721	0.463	0.3 to 1.728	0.190	127	0.639	0.318	0.265 to 1.54
DQB1*03:03	0.196	9	0.1128	106	1.913	0.093	0.898 to 4.074	0.123	82	1.738	0.157	0.809 to 3.731
DQB1*05:01	0.109	5	0.1404	132	0.747	0.545	0.29 to 1.924	0.078	52	1.446	0.457	0.548 to 3.816
DQB1*05:02	0.370	17	0.2128	200	2.169	0.014	1.168 to 4.026	0.184	123	2.598	0.003	1.384 to 4.877

DQB1*05:03	0.065	3	0.0404	38	1.657	0.415	0.492 to 5.583	0.045	30	1.484	0.528	0.435 to 5.059
DQB1*06:01	0.087	4	0.0713	67	1.240	0.689	0.432 to 3.564	0.093	62	0.931	0.895	0.323 to 2.683
DRB1*09:01	0.174	8	0.0989	93	1.918	0.107	0.869 to 4.235	0.108	72	1.742	0.174	0.782 to 3.88
DRB1*12:02	0.130	6	0.1532	144	0.829	0.675	0.345 to 1.991	0.148	99	0.862	0.742	0.356 to 2.087
DRB1*14:04	0.065	3	0.0234	22	2.912	0.092	0.839 to 10.107	0.024	16	2.837	0.108	0.796 to 10.113
DRB1*15:01	0.283	13	0.0809	76	4.476	0.000	2.26 to 8.863	0.085	57	4.224	0.000	2.104 to 8.48
DRB1*15:02	0.152	7	0.1447	136	1.060	0.888	0.465 to 2.42	0.105	70	1.533	0.320	0.661 to 3.558

*Yellow highlight indicates statistically significant association
