# Supplementary material for: Human Leukocyte Antigen Markers for Distinguishing Pustular Psoriasis and Adult-Onset Immunodeficiency with Pustular Reaction
Source: Genes (Basel). 2024 Feb 23;15(3):278. doi: 10.3390/genes15030278 (PMC10970016; doi:10.3390/genes15030278)
Supplement: Supplementary file 1 [file genes-15-00278-s001.zip › TableS5.pdf]

Table S5 Enriched mutations that cause amino acid change in HLA genes in patients with pustular skin diseases

No	RS ID	Gene	Type*	Position	ALT	Amino acid change	Binding pocket	Pustular lesion patients in Thailand			1000G EAS					gnomAD EAS				
								AC	AN	AF	AC	AN	AF	P	adj P	AC	AN	AF	P	adj P
1	rs1051488	B	M	31355134-31355134	T	p.Ala329Thr	N	47	82	0.573	296	992	0.298	7.0E-07	1.7E-04	102	1500	0.068	5.6E-30	1.4E-27
2	rs1131500	B	M	31355203-31355203	T	p.Val306Ile	N	47	82	0.573	238	920	0.259	9.8E-09	2.4E-06	83	1324	0.063	7.9E-31	1.9E-28
3	rs12697943	B	M	31356280-31356280	A	p.Arg169Leu	Y	16	82	0.195	61	1146	0.053	1.9E-05	4.6E-03	19	2144	0.009	6.0E-15	1.5E-12
4	rs1050654	B	M	31356323-31356323	T	p.Arg155Ser	N	72	82	0.878	487	1074	0.453	9.2E-15	2.2E-12	196	1288	0.152	7.3E-44	1.8E-41
5	rs4997052	B	M	31356367-31356367	G	p.Tyr140Ser	Y	36	82	0.439	142	1002	0.142	7.0E-10	1.7E-07	66	1268	0.052	1.3E-21	3.1E-19
6	rs12721829	B	M	31356431-31356431	T	p.Leu119Ile	Y	32	82	0.390	94	930	0.101	9.8E-11	2.4E-08	16	1048	0.015	5.3E-28	1.3E-25
7	rs12721827	B	M	31356433-31356433	A	p.Thr118Ile	Y	32	82	0.390	93	952	0.098	4.3E-11	1.1E-08	13	1378	0.009	5.6E-33	1.4E-30
8	rs3180380	B	M	31356712-31356712	A	p.Gly107Cys	Y	21	82	0.256	77	922	0.084	1.0E-05	2.6E-03	58	1306	0.044	6.4E-10	1.6E-07
9	rs1050388	B	M	31356729-31356729	T	p.Ser101Asn	Y	33	82	0.402	206	1044	0.197	3.7E-05	9.0E-03	128	1374	0.093	1.1E-12	2.7E-10
10	rs1131215	B	M	31356739-31356739	A	p.Asp98Tyr	Y	63	82	0.768	546	984	0.555	9.1E-05	2.2E-02	495	1116	0.444	7.3E-09	1.8E-06
11	rs1050538	B	M	31356822-31356822	G	p.Glu70Ala	N	42	82	0.512	79	1012	0.078	8.6E-22	2.1E-19	24	1512	0.016	1.1E-42	2.6E-40
12	rs9266193	B	M	31357011-31357011	C	p.Glu18Gly	N	63	82	0.768	480	976	0.492	7.5E-07	1.8E-04	155	730	0.212	1.4E-23	3.5E-21
13	rs9266197	B	M	31357053-31357053	A	p.Ala4Val	N	76	82	0.927	660	932	0.708	2.4E-06	5.9E-04	177	752	0.235	3.6E-36	8.7E-34
14	rs1131165	B	M	31357110-31357110	C	p.Leu17Val	N	42	82	0.512	246	970	0.254	1.5E-06	3.7E-04	31	1004	0.031	4.7E-33	1.2E-30
15	rs1131159	B	M	31357115-31357115	C	p.Ala15Gly	N	69	82	0.841	431	908	0.475	3.8E-11	9.3E-09	110	804	0.137	3.1E-40	7.6E-38
16	rs1050458	B	M	31357148-31357148	G	p.Met4Thr	N	76	82	0.927	632	914	0.691	6.0E-07	1.5E-04	188	726	0.259	1.7E-33	4.1E-31
17	rs9266206	B	M	31357154-31357154	C	p.Leu2Arg	N	76	82	0.927	633	906	0.699	1.1E-06	2.7E-04	199	718	0.277	1.2E-31	3.0E-29
18	rs707908	C	M	31270276-31270276	C	p.Gln277Glu	N	61	82	0.744	10	420	0.024	2.1E-49	5.2E-47	1	1392	0.001	1.1E-88	2.6E-86
19	rs1050276	C	M	31270291-31270291	T	p.Val272Met	N	11	82	0.134	5	590	0.008	1.3E-07	3.1E-05	1	1572	0.001	2.6E-14	6.4E-12
20	rs1050328	C	M	31270378-31270378	A	p.Arg243Trp	N	47	82	0.573	23	558	0.041	1.5E-31	3.6E-29	4	1462	0.003	2.9E-62	7.1E-60
21	rs1050716	C	M	31270453-31270453	C	p.Leu218Val	N	61	82	0.744	43	470	0.091	7.8E-35	1.9E-32	5	1450	0.003	1.3E-84	3.2E-82
22	rs1131096	C	M	31270482-31270482	T	p.Pro208His	N	61	82	0.744	38	460	0.083	3.2E-36	7.9E-34	4	1440	0.003	1.5E-85	3.7E-83
23	rs2308590	C	M	31271165-31271165	T	p.Ala176Glu	Y	53	82	0.646	200	674	0.297	9.0E-10	2.2E-07	18	1204	0.015	5.3E-57	1.3E-54
24	rs1050366	C	M	31271180-31271180	C	p.Leu171Trp	Y	61	82	0.744	380	770	0.494	9.3E-06	2.3E-03	66	1158	0.057	2.7E-49	6.7E-47
25	rs713032	C	M	31271273-31271273	A	p.Ser140Phe	Y	24	82	0.293	37	786	0.047	3.8E-11	9.2E-09	67	1400	0.048	8.1E-12	2.0E-09
26	rs713032	C	M	31271273-31271273	T	p.Ser140Tyr	Y	32	82	0.390	82	786	0.104	3.1E-10	7.6E-08	17	1392	0.012	3.2E-31	7.9E-29

27	rs2308575	C	M	31271280-31271280	T	p.Asp138Asn	Y	16	82	0.195	24	926	0.026	1.1E-08	2.8E-06	5	1634	0.003	2.8E-18	6.9E-16
28	rs34592426	C	M	31271313-31271313	C	p.Leu127Val	N	23	82	0.280	61	944	0.065	1.6E-08	4.0E-06	6	1584	0.004	1.2E-26	3.0E-24
29	rs1131115	C	M	31271324-31271324	T	p.Ser123Tyr	Y	47	82	0.573	193	794	0.243	1.8E-09	4.4E-07	145	1232	0.118	7.1E-21	1.7E-18
30	rs1071649	C	M	31271337-31271337	T	p.Leu119Ile	Y	23	82	0.280	45	912	0.049	2.6E-10	6.4E-08	5	1556	0.003	3.9E-27	9.5E-25
31	rs1131119	C	M	31271339-31271339	A	p.Thr118Ile	Y	25	82	0.305	54	902	0.060	2.3E-10	5.6E-08	5	1552	0.003	7.9E-30	1.9E-27
32	rs1131123	C	M	31271601-31271601	G	p.Asp114Ala	N	52	82	0.634	368	950	0.387	1.3E-05	3.2E-03	258	1352	0.191	3.4E-17	8.3E-15
33	rs707911	C	M	31271800-31271800	C	p.Ser48Ala	Y	49	82	0.598	336	1040	0.323	8.8E-07	2.2E-04	297	1856	0.160	3.9E-18	9.5E-16
34	rs1050437	C	M	31271808-31271808	T	p.Arg45His	N	31	82	0.378	140	1084	0.129	5.9E-08	1.4E-05	90	2398	0.038	2.2E-21	5.5E-19
35	rs1126504	DPB1	M	33080680-33080680	G	p.Leu37Val	Y	36	82	0.439	268	1170	0.229	4.3E-05	1.0E-02	1130	5174	0.218	8.3E-06	2.0E-03
36	rs1126509	DPB1	M	33080684-33080684	A	p.Phe38Tyr	Y	36	82	0.439	268	1170	0.229	4.3E-05	1.0E-02	1130	5164	0.219	8.7E-06	2.1E-03
37	rs1126513	DPB1	M	33080690-33080690	T	p.Gly40Val	Y	34	82	0.415	265	1170	0.226	2.0E-04	4.9E-02	1073	5186	0.207	1.9E-05	4.7E-03
38	rs1042117	DPB1	M	33080762-33080762	A	p.Phe64Tyr	Y	22	82	0.268	101	1170	0.086	3.8E-06	9.3E-04	394	5246	0.077	1.6E-07	3.8E-05
39	rs1126537	DPB1	M	33084959-33084959	A	p.Arg125Lys	N	60	82	0.732	103	650	0.158	4.3E-26	1.1E-23	315	1124	0.280	5.1E-16	1.3E-13
40	rs1130432	DQB1	M	32661352-32661352	C	p.Gln256Arg	N	47	81	0.580	274	1168	0.235	1.6E-10	4.0E-08	884	4340	0.204	2.6E-13	6.3E-11
41	rs41542812	DQB1	M	32662154-32662154	G	p.Gln158His	N	30	82	0.366	133	1170	0.114	1.4E-08	3.4E-06	420	3674	0.114	5.1E-09	1.2E-06
42	rs1049107	DQB1	M	32662159-32662159	T	p.Gly157Ser	N	46	82	0.561	268	1170	0.229	5.1E-10	1.2E-07	860	3670	0.234	3.6E-10	8.8E-08
43	rs1049100	DQB1	M	32662186-32662186	T	p.Val148Ile	N	46	82	0.561	276	1168	0.236	1.4E-09	3.4E-07	860	3696	0.233	2.8E-10	6.9E-08
44	rs17412833	DQB1	M	32664821-32664821	T	p.Phe119Tyr	Y	51	82	0.622	371	1160	0.320	6.1E-08	1.5E-05	1178	3910	0.301	3.0E-09	7.5E-07
45	rs1130392	DQB1	M	32664851-32664851	C	p.Thr109Arg	Y	49	82	0.598	301	1164	0.259	5.1E-10	1.3E-07	927	4346	0.213	1.0E-13	2.6E-11
46	rs9274384	DQB1	M	32664858-32664858	C	p.Leu107Val	Y	50	82	0.610	335	1160	0.289	6.3E-09	1.5E-06	987	4070	0.243	3.2E-12	7.9E-10
47	rs1130387	DQB1	M	32664860-32664860	G	p.Glu106Ala	Y	50	82	0.610	334	1160	0.288	5.7E-09	1.4E-06	996	4116	0.242	3.0E-12	7.3E-10
48	rs1130390	DQB1	M	32664870-32664870	C	p.Thr103Ala	Y	47	82	0.573	293	1146	0.256	4.9E-09	1.2E-06	921	3960	0.233	6.4E-11	1.6E-08
49	rs1071637	DQB1	M	32664911-32664911	C	p.Asp89Gly	Y	31	82	0.378	136	1168	0.116	5.8E-09	1.4E-06	451	4616	0.098	2.2E-11	5.5E-09
50	rs41552812	DQB1	M	32664912-32664912	T	p.Asp89Asn	Y	31	82	0.378	136	1170	0.116	5.6E-09	1.4E-06	450	4766	0.094	9.4E-12	2.3E-09
51	rs1063318	DQB1	M	32664968-32664968	A	p.Ala70Val	Y	55	82	0.671	470	1170	0.402	1.8E-06	4.5E-04	1667	4580	0.364	2.3E-08	5.6E-06
52	rs281862065	DQB1	M	32664993-32664993	G	p.Tyr62His	Y	46	82	0.561	300	1170	0.256	1.8E-08	4.4E-06	1023	4620	0.221	4.5E-11	1.1E-08
53	rs1049066	DQB1	M	32665004-32665004	C	p.Leu58Arg	Y	48	82	0.585	350	1170	0.299	2.1E-07	5.2E-05	1262	4646	0.272	3.6E-09	8.7E-07
54	rs12722115	DQB1	M	32665005-32665005	C	p.Leu58Val	Y	48	82	0.585	350	1170	0.299	2.1E-07	5.2E-05	1260	4630	0.272	3.8E-09	9.3E-07
55	rs1130368	DQB1	M	32665041-32665041	G	p.Met46Leu	Y	46	82	0.561	272	1170	0.232	8.2E-10	2.0E-07	871	4738	0.184	5.4E-14	1.3E-11
56	rs1049059	DQB1	M	32666536-32666536	G	p.Met24Ile	N	46	82	0.561	262	1170	0.224	2.4E-10	6.0E-08	892	5082	0.176	9.1E-15	2.2E-12

57	rs1049056	DQB1	M	32666592-32666592	A	p.Ala6Ser	N	46	82	0.561	262	1170	0.224	2.4E-10	6.0E-08	891	5062	0.176	1.0E-14	2.5E-12
58	rs9269744	DRB1	M	32580249-32580249	C	p.Thr262Arg	N	16	76	0.211	42	1100	0.038	1.8E-07	4.4E-05	32	2414	0.013	1.2E-13	2.9E-11
59	rs77637983	DRB1	M	32581675-32581675	G	p.Gln178His	N	18	82	0.220	12	1140	0.011	5.3E-15	1.3E-12	35	3166	0.011	8.1E-17	2.0E-14
60	rs17885482	DRB1	M	32584135-32584135	C	p.Val115Gly	Y	47	82	0.573	139	1090	0.128	2.1E-19	5.2E-17	316	2654	0.119	9.4E-22	2.3E-19
61	rs17886918	DRB1	M	32584193-32584193	A	p.Ile96Phe	Y	19	82	0.232	40	1122	0.036	1.4E-09	3.4E-07	43	2204	0.020	5.2E-14	1.3E-11
62	rs16822820	DRB1	M	32584283-32584283	T	p.Ser66Thr	Y	14	82	0.171	43	1170	0.037	6.9E-06	1.7E-03	145	4278	0.034	1.1E-06	2.6E-04
63	rs3175105	DRB1	M	32584303-32584303	C	p.Tyr59Cys	Y	12	82	0.146	23	1168	0.020	7.0E-07	1.7E-04	68	4648	0.015	7.5E-09	1.8E-06
64	rs17879746	DRB1	M	32589645-32589645	T	p.Arg33Gln	N	14	82	0.171	52	1144	0.045	5.4E-05	1.3E-02	39	1984	0.020	5.4E-09	1.3E-06
65	rs9270302	DRB1	M	32589702-32589702	A	p.Ala14Val	N	18	82	0.220	7	1116	0.006	5.5E-17	1.3E-14	2	2170	0.001	3.2E-25	8.0E-23
66	rs375356947	B	F	31356711-31356711	GG	p.Gly107AlafsTer45	Y	21	82	0.256	77	1124	0.069	5.2E-07	1.3E-04	53	1898	0.028	1.8E-13	4.5E-11
67	rs576010607	B	F	31356716-31356717	-	p.Leu105ArgfsTer46	Y	21	82	0.256	78	1116	0.070	6.9E-07	1.7E-04	57	1612	0.035	1.2E-11	2.9E-09
68	rs66473235	B	F	31356719-31356720	-	p.Asn104ThrfsTer47	Y	21	82	0.256	78	1114	0.070	7.1E-07	1.7E-04	60	1558	0.039	4.8E-11	1.2E-08
69	rs750527298	B	F	31356747-31356749	-	p.Gln94HisfsTer4	Y	62	82	0.756	555	1088	0.510	9.0E-06	2.2E-03	528	1308	0.404	3.4E-10	8.4E-08
70	rs9281379	B	F	31356824-31356824	A	p.Glu69AspfsTer30	N	42	82	0.512	80	1118	0.072	3.3E-23	8.0E-21	24	1582	0.015	1.8E-43	4.3E-41
71	rs200186034	B	F	31356826-31356827	-	p.Glu69ArgfsTer8	N	62	82	0.756	365	960	0.380	3.5E-11	8.6E-09	205	1120	0.183	8.5E-27	2.1E-24
72	rs67523850	DPB1	F	33086236-33086237	-	p.Lys232AsnfsTer14	N	21	82	0.256	102	1170	0.087	1.5E-05	3.6E-03	382	5188	0.074	5.0E-07	1.2E-04
73	rs753527747	DQB1	F	32666524-32666526	-	p.Leu28ThrfsTer3	N	13	82	0.159	29	1170	0.025	8.6E-07	2.1E-04	86	2434	0.035	1.2E-05	2.8E-03
74	rs778891121	DQB1	F	32666527-32666527	AA	p.Leu28SerfsTer33	N	13	82	0.159	29	1170	0.025	8.6E-07	2.1E-04	86	5112	0.017	3.0E-09	7.3E-07
75	rs67187877	DRB1	F	32584174-32584176	-	p.Ala102ArgfsTer25	Y	17	82	0.207	49	1032	0.047	1.5E-06	3.6E-04	13	2786	0.005	9.1E-20	2.2E-17
76	rs28986201	DRB1	F	32584184-32584184	T	p.Gln99ThrfsTer29	Y	18	82	0.220	53	1100	0.048	3.7E-07	8.9E-05	35	2818	0.012	5.7E-16	1.4E-13
77	rs764153503	DRB1	F	32584185-32584185	T	p.Gln99AlafsTer29	Y	18	82	0.220	53	1102	0.048	3.6E-07	8.7E-05	36	2960	0.012	3.7E-16	9.0E-14

* M= missense, F= Frameshift mutation

* AN = Allele count, AN = Total allele, AF = Allele frequency

* P = P-value of hypergeometric test, adj P = Bonferroni adjusted P-value for multiple comparisons
