# Supplementary material for: Human Leukocyte Antigen Markers for Distinguishing Pustular Psoriasis and Adult-Onset Immunodeficiency with Pustular Reaction
Source: Genes (Basel). 2024 Feb 23;15(3):278. doi: 10.3390/genes15030278 (PMC10970016; doi:10.3390/genes15030278)
Supplement: Supplementary file 1 [file genes-15-00278-s001.zip › TableS6.pdf]

Table S6 Odd ratio of HLA alleles in pustular reaction in AOID patients in comparison with pustular psoriasis patient control

HLA allele	AOID with pustular reaction		PP		Odds ratio	95% CI	P-value
	2n=36	AF	2n=46	AF			
A*33:03	0	0.000	4	0.087	NA	NA	NA
A*02:03	8	0.222	4	0.087	3.385	0.928 to 12.351	0.065
A*02:06	2	0.056	1	0.022	2.938	0.255 to 33.775	0.387
A*02:07	2	0.056	9	0.196	0.271	0.055 to 1.344	0.110
A*11:01	10	0.278	13	0.283	1.122	0.423 to 2.972	0.817
A*11:02	3	0.083	1	0.022	4.548	0.452 to 45.74	0.198
A*24:02	4	0.111	5	0.109	1.147	0.284 to 4.627	0.848
A*24:07	3	0.083	3	0.065	1.452	0.275 to 7.668	0.661
A*24:10	1	0.028	3	0.065	0.455	0.045 to 4.567	0.503
B*13:01	7	0.194	9	0.196	1.123	0.373 to 3.385	0.836
B*15:02	2	0.056	3	0.065	0.938	0.148 to 5.937	0.945
B*15:25	1	0.028	3	0.065	0.455	0.045 to 4.567	0.503
B*18:01	1	0.028	2	0.043	0.697	0.061 to 8.011	0.772
B*18:02	2	0.056	2	0.043	1.438	0.192 to 10.742	0.724
B*38:02	2	0.056	1	0.022	2.938	0.255 to 33.775	0.387
B*40:01	5	0.139	2	0.043	3.966	0.721 to 21.804	0.113
B*46:01	3	0.083	10	0.217	0.368	0.093 to 1.454	0.154
B*51:01	1	0.028	4	0.087	0.333	0.036 to 3.123	0.336
C*01:02	1	0.028	10	0.217	0.115	0.014 to 0.948	0.044*
C*03:04	11	0.306	9	0.196	2.072	0.747 to 5.751	0.162
C*04:03	1	0.028	3	0.065	0.455	0.045 to 4.567	0.503
C*07:02	6	0.167	5	0.109	1.843	0.513 to 6.62	0.349
C*07:04	3	0.083	3	0.065	1.452	0.275 to 7.668	0.661
C*08:01	3	0.083	5	0.109	0.832	0.185 to 3.745	0.811
C*14:02	1	0.028	4	0.087	0.333	0.036 to 3.123	0.336
DPB1*02:01	3	0.083	10	0.217	0.368	0.093 to 1.454	0.154
DPB1*02:02	2	0.056	3	0.065	0.938	0.148 to 5.937	0.945
DPB1*03:01	2	0.056	1	0.022	2.938	0.255 to 33.775	0.387
DPB1*05:01	16	0.444	9	0.196	3.852	1.432 to 10.36	0.008
DPB1*13:01	6	0.167	12	0.261	0.643	0.215 to 1.926	0.430
DPB1*14:01	1	0.028	4	0.087	0.333	0.036 to 3.123	0.336
DPB1*21:01	0	0.000	3	0.065	NA	NA	NA
DQB1*03:01	1	0.028	6	0.130	0.212	0.024 to 1.85	0.160
DQB1*03:03	4	0.111	9	0.196	0.578	0.162 to 2.058	0.397
DQB1*05:01	8	0.222	5	0.109	2.646	0.782 to 8.953	0.118
DQB1*05:02	13	0.361	17	0.370	1.129	0.454 to 2.805	0.794
DQB1*05:03	0	0.000	3	0.065	NA	NA	NA
DQB1*06:01	3	0.083	4	0.087	1.065	0.222 to 5.096	0.938
DRB1*04:05	3	0.083	0	0.000	NA	NA	NA
DRB1*09:01	4	0.111	8	0.174	0.667	0.183 to 2.422	0.538
DRB1*12:02	1	0.028	6	0.130	0.212	0.024 to 1.85	0.160
DRB1*14:04	0	0.000	3	0.065	NA	NA	NA
DRB1*15:01	4	0.111	13	0.283	0.359	0.106 to 1.219	0.100
DRB1*15:02	12	0.333	7	0.152	3.195	1.1 to 9.281	0.033
DRB1*16:02	9	0.250	0	0.000	NA	NA	NA

Abbreviation: AOID, adult-onset immunodeficiency; AF, allele frequency; PP, pustular psoriasis; CI, confidence interval

* HLA-C*01:02 was statistically significant in this comparison, but there was no association between this allele and pustular reaction when compared to healthy controls.
