# Supplementary material for: Human Leukocyte Antigen Markers for Distinguishing Pustular Psoriasis and Adult-Onset Immunodeficiency with Pustular Reaction
Source: Genes (Basel). 2024 Feb 23;15(3):278. doi: 10.3390/genes15030278 (PMC10970016; doi:10.3390/genes15030278)
Supplement: Supplementary file 1 [file genes-15-00278-s001.zip › TableS7.pdf]

Table S7 Linkage disequilibrium analysis based on r^2 metric of AOID-associated SNVs

**SNV**	**rs9269744**	**rs77637983**	**rs17885482**	**rs9270302**
**rs9269744**	1			
**rs77637983**	0.868	1		
**rs17885482**	0.287	0.417	1	
**rs9270302**	0.868	1	0.417	1
