# Supplementary material for: Human Leukocyte Antigen Markers for Distinguishing Pustular Psoriasis and Adult-Onset Immunodeficiency with Pustular Reaction
Source: Genes (Basel). 2024 Feb 23;15(3):278. doi: 10.3390/genes15030278 (PMC10970016; doi:10.3390/genes15030278)
Supplement: Supplementary file 1 [file genes-15-00278-s001.zip › TableS8.pdf]

Table S8 Association between HLA variants and DRB1 alleles

**location**	**Alt**	**Accession ID**	***DRB1:1501***		***DRB1:1502***		***DRB1:1602***	
			**Odds**	**P**	**Odds**	**P**	**Odds**	**P**
6:32580249-32580249	C	rs9269744	1.227	0.769	0.381	0.266	NA	NA
6:32581675-32581675	G	rs77637983	2.21	0.253	0.222	0.093	NA	NA
6:32584135-32584135	C	rs17885482	25.93	0.002	0.158	0.006	0.22	0.058
6:32589702-32589702	A	rs9270302	2.21	0.253	0.222	0.093	NA	NA
